# Improvement of the Piezoresistive Behavior of Poly (vinylidene fluoride)/Carbon Nanotube Composites by the Addition of Inorganic Semiconductor Nanoparticles

**DOI:** 10.3390/ma17040774

**Published:** 2024-02-06

**Authors:** Müslüm Kaplan, Emre Alp, Beate Krause, Petra Pötschke

**Affiliations:** 1 Faculty of Engineering, Architecture and Design, Bartin University, Bartin 74110, Turkey; mkaplan@bartin.edu.tr (M.K.); emrealp@bartin.edu.tr (E.A.); 2 Leibniz-Institut für Polymerforschung Dresden e.V. (IPF), Hohe Str. 6, 01069 Dresden, Germany; krause-beate@ipfdd.de

**Keywords:** poly (vinylidene fluoride) (PVDF), strain sensing, multi-wall carbon nanotubes (MWCNTs), inorganic semiconductor, nanomaterials, piezoresistivity

## Abstract

Conductive polymer composites (CPCs), obtained by incorporating conductive fillers into a polymer matrix, are suitable for producing strain sensors for structural health monitoring (SHM) in infrastructure. Here, the effect of the addition of inorganic semiconductor nanoparticles (INPs) to a poly (vinylidene fluoride) (PVDF) composite filled with multi-walled carbon nanotubes (MWCNTs) on the piezoresistive behavior is investigated. INPs with different morphologies and sizes are synthesized by a hydrothermal method. The added inorganic oxide semiconductors showed two distinct morphologies, including different phases. While particles with flower-like plate morphology contain phases of orth-ZnSnO_3_ and SnO, the cauliflower-like nanoparticles contain these metal oxides and ZnO. The nanoparticles are characterized by field-emission scanning electron microscopy (FE-SEM) and X-ray diffraction (XRD), and the nanocomposites by Fourier transform infrared spectroscopy (FTIR), scanning electron microscopy (SEM), differential scanning calorimetry (DSC), and thermogravimetric analysis (TGA). Cyclic tensile testing is applied to determine the strain-sensing behavior of PVDF/1 wt% MWCNT nanocomposites with 0–10 wt% inorganic nanoparticles. Compared to the PVDF/1 wt% MWCNT nanocomposite, the piezoresistive sensitivity is higher after the addition of both types of nanoparticles and increases with their amount. Thereby, nanoparticles with flower-like plate structures improve strain sensing behavior slightly more than nanoparticles with cauliflower-like structures. The thermogravimetric analysis results showed that the morphology of the semiconductor nanoparticles added to the PVDF/MWCNT matrix influences the changes in thermal properties.

## 1. Introduction

The demand for high-performance, adaptable, intelligent, and flexible strain sensors is increasing. Conductive polymer composites (CPCs), utilizing the stimulus-response behavior of conductive networks to environmental conditions, can be designed as sensors to monitor and/or detect temperature variations, damage conditions, strain/stress changes, and the existence of liquids or vapors in the environment [1,2]. CPC-based strain sensors have recently attracted increasing attention due to their light weight, flexibility, stretchability, and easy processing [3]. They have great potential in various applications, including human movement detection [4], human-machine interfaces [5], wearable health monitoring solutions [6], soft robotic skins [7], artificial muscles [8], and structural health monitoring [9].

CPC-based strain sensors, which are generated by the incorporation of conductive fillers into an insulating polymer matrix, can be produced with a wide range of polymers as matrices, including elastomers/duromers, and thermoplastics, to fulfill application necessities [10,11]. Poly(vinylidene fluoride) (PVDF) [12], polypropylene [13], polycarbonate [14], polystyrene [15], and polyethylene [16] have recently attracted attention to exploiting the piezoresistive behavior of CPCs with thermoplastic polymer matrices. Among them, PVDF attracts attention because of its good environmental resistance, excellent flexibility, high strength, easy processing, and low cost, as well as its excellent mechanical properties compared to other semi-crystalline and amorphous thermoplastics [17,18,19]. Additionally, PVDF is intriguing for constructing strain sensors because of its piezoelectric characteristics. The piezoelectric property of PVDF is due to polar crystalline phases, including the γ phase and β phase, which show the most significant electric dipole moment among all PVDF crystalline phases [20,21]. Although PVDF exhibits properties that make it a promising candidate for self-powered sensors, strain sensors, and energy harvesting devices [18,19], its low piezoelectric coefficient limits its strain-sensing capabilities. Therefore, numerous studies have been conducted to improve the piezoresistive properties of PVDF-based CPCs by using different nanofillers and their synergistic effects [22,23,24,25].

Among the conductive fillers used to build sensitive conductive networks, multi-walled carbon nanotubes (MWCNTs) with high aspect ratios are advantageous because only low amounts are needed to achieve electrical filler percolation [26]. The electrical resistance–strain sensitivity of polymer/MWCNT nanocomposites has been explored in various aspects, including using elastomers/rubbers with high elasticity as a matrix [27], functionalized MWCNTs [28], the application of interfacial compatibilizers [29], and tuning the network structure with the addition of second fillers [30,31].

Inorganic semiconductor nanoparticles (INPs) such as ZnO [32,33,34], ZnSnO_3_ [35,36], and ZnS [37,38] have attracted considerable attention in recent years due to their piezoresistive and piezoelectric properties combined with environmental friendliness. The incorporation of these INPs, which generate an electric charge when subjected to mechanical stress, in combination with highly conductive MWCNTs in PVDF seems to be a favorable combination to improve the piezoresistive behavior. Moreover, since the piezoresistive coefficient is known to increase as the scale of materials decreases [39], the fact that INPs can be synthesized at the nanoscale may also contribute to improvements in sensitivity. Thus, from the combination of percolated networks of MWCNTs with nanosized INPs, an increase in the piezoresistive response was expected.

In this contribution, the strategy of combining MWCNTs and semiconductor INPs with different morphologies, sizes, and phases as a hybrid filler system to generate PVDF-based susceptible piezoresistive strain sensor materials is presented. To our best knowledge, such combinations were not studied before in melt-mixed composites. For the synthesis of the INPs, mixed phases of metal oxides were aimed at because they are likely to exhibit unusual properties not found in their single phases. The added INPs had two distinct morphologies, including different phases. One type has flower-like plate nanoparticles, having orth-ZnSnO_3_ and SnO. The other has cauliflower-like nanoparticles, which have ZnO in addition to these metal oxides. The effect of adding these inorganic semiconductors to PVDF/MWCNT composites on their electrical resistance change ΔR/R_0_ under cycling strain and their thermal stability was examined. It was shown that the piezoresistive behavior of the PVDF/MWCNT-based nanocomposites under cyclic loading conditions was improved by embedding these as-synthesized nanocrystals.

## 2. Experimental Section

### 2.1. Materials and Sample Preparation

#### 2.1.1. Synthesis of Inorganic Nanoparticles (INPs)

The chemicals used were of analytical grade and were not subjected to any further purification. Two types of inorganic nanoparticles (INPs) were synthesized in a one-step procedure via the hydrothermal method. To produce one (INPs1), 0.75 g zinc acetate dihydrate, 0.75 g tin sulfate, and 0.75 g sodium hydroxide were dissolved in 50 mL of ultrapure water under magnetic stirring. The obtained homogenous solution was sealed in a 100 mL Teflon-lined stainless-steel autoclave. Then, the Teflon-lined stainless-steel autoclave was maintained for 12 h at 160 °C. Similarly, another type of INP (INPs2) was synthesized by dissolving 0.75 g zinc acetate dihydrate, 0.75 g tin sulfate, and 0.75 g sodium hydroxide in 50 mL of ultrapure water and maintaining it for 12 h at 120 °C. All hydrothermally synthesized nanoparticles were dried in an oven at 60 °C overnight after cleaning. These as-synthesized INPs were embedded into a PVDF/1 wt% MWCNT composite material in different amounts to improve its piezoresistive-strain sensing behavior.

#### 2.1.2. Production of PVDF/MWCNT/INP Composites

PVDF, specifically Kynar™ 720 procured from Arkema Inc., Paris, France, was employed as the polymer matrix. Non-functionalized, short-thin MWCNTs designated as NC3150 were used as conductive fillers. MWCNTs with around 1 micrometer and an average outer diameter of 9.5 nanometers were procured from Nanocyl, S.A., Sambreville, Belgium. The PVDF/1 wt% MWCNT/nanocrystal composites were fabricated through a one-step melt-mixing process using a twin-screw microcompounder (Xplore, Geleen, The Netherlands) with a 15 cm^3^ capacity at a mass temperature of 210 °C, a rotation speed of 200 rpm, and a mixing time of 10 min. Before mixing, PVDF pellets were dried at 120 °C, and the nanocrystals and CNTs were dried at 85 °C for 12 h in a vacuum oven. The extruded strands were cut into small pieces and dried at 120 °C for 12 h in a vacuum oven before being compression molded into rectangular plates of 80 mm × 55 mm × 0.5 mm using a hot press (Model-PW40EH, Paul-Otto Weber GmbH, Remshalden, Germany) at a set temperature of 210 °C. The samples were preheated for 1 min and then pressed under a closing force of 50 kN for 2.5 min. After pressing, the samples are cooled from 210 °C to room temperature within 30 s using an accessory water-cooling device.

The compression-molded rectangular samples were cut into dumbbell-shaped specimens with a length of 7.5 cm and a narrow parallel section of 25 mm in length and 4 mm in width according to the DIN EN ISO 527-2 5A standard [40] for mechanical and piezoresistive tests.

### 2.2. Characterization

Phase characteristics of the as-synthesized INPs were studied by a RIGAKU SmartLab™ X-ray diffractometer (XRD) (Rigaku Corporation, Tokyo, Japan) operated at 40 mA current and 40 kV voltage with Cu-Kα radiation (λ = 1.5406 Å). The microstructural and morphological properties of the INPs were determined via a TESCAN™ MAIA XMU field emission gun scanning electron microscope (FESEM) (TESCAN, Brno, Czech Republic).

The state of MWCNT and INP macrodispersion in the nanocomposites was analyzed by light transmission microscopy. Thin sections of 5 μm in thickness were prepared perpendicular to the direction of the extruded strand using a Leica RM 2265 microtome from Leica Microsystems GmbH, Wetzlar, Germany. The cuts were embedded on glass slides using Aquatex^®^ medium (Merck, Darmstadt, Germany). The samples were then characterized using a BH2 microscope in transmission mode combined with a DP74 camera from Olympus Deutschland GmbH, Hamburg, Germany. The Ultra Plus scanning electron microscope (SEM) (Zeiss, Oberkochen, Germany) was used to image the cryo-fractured surface of composite materials at a beam energy of 3 kV. Before obtaining SEM images, the surfaces of the composites were made conductive with a 3 nm platin coating.

The hysteresis piezoresistivity measurements of PVDF/MWCNT/INP composites were performed according to DIN EN 527-2/S2/1 on a universal testing machine 1456 Zwick (Zwick Roell, Ulm, Germany) connected with a Keithley 2001 electrometer. The equipment simultaneously records the mechanical behavior and electrical resistance changes of the samples under uniaxial stretching. The samples underwent a cross-head speed of 1 mm/min and had an initial clamping distance of 30 mm. 0.5 N of preload was used for the tensile test. In order to lower the contact resistance between the electrode clamps and the specimen, two silver paste marks were painted on the surface of the dog-bone specimens adjacent to the end of the parallel segment before the testing. The strain values shown are not precise and somewhat underestimated, assuming that the elongation primarily occurs in the specimen’s parallel section (which has a length of 25 mm). The relative resistance was measured as ΔR/R_0_, where R_0_ represents the initial electrical resistance following the sample’s clamping in the sample holders, and ΔR represents the change in electrical resistance. The cyclic testing was carried out at 1 mm/min speed with a maximum loading strain of 3% for 15 cycles. The samples were held for 90 s at the end of loading and unloading to relieve the residual stress before the next cycle was performed.

Fourier-transform infrared (FTIR) spectra of the composites were obtained using a Vertex 80v spectrometer (Bruker, Billerica, MA, USA) using a golden gate diamond ATR unit (SPECAC), with 100 scans with a resolution of 4 cm^−1^ in the range between 4000–600 cm^−1^.

The PVDF/MWCNT/INP composites were analyzed by differential scanning calorimetry (DSC) and thermal gravimetric analysis (TGA) to determine the thermal properties following ASTM D3418 [41] and ASTM E1131 [42], respectively. TGA was performed under a nitrogen atmosphere using a TGA Q5000 (TA Instruments-Waters LLC, New Castle, DE, USA) with a 10 K/min heating ramp from 0 to 800 °C. The DSC analysis of the composites was performed with DSC2500 (TA Instrument-Waters LLC, New Castle, DE, USA). The sample was initially equilibrated at −110 °C for 5 min. It was then heated at a rate of 10 K/min to 200 °C and maintained at this temperature for 30 s. The sample was cooled at 10 K/min to −110 °C and held at this temperature for 5 min. Finally, the sample was reheated at 10 K/min to 200 °C. This protocol allowed for the comprehensive study of the thermal properties and behavior of the composites under varying temperature conditions.

## 3. Results and Discussions

**Figure 1:**
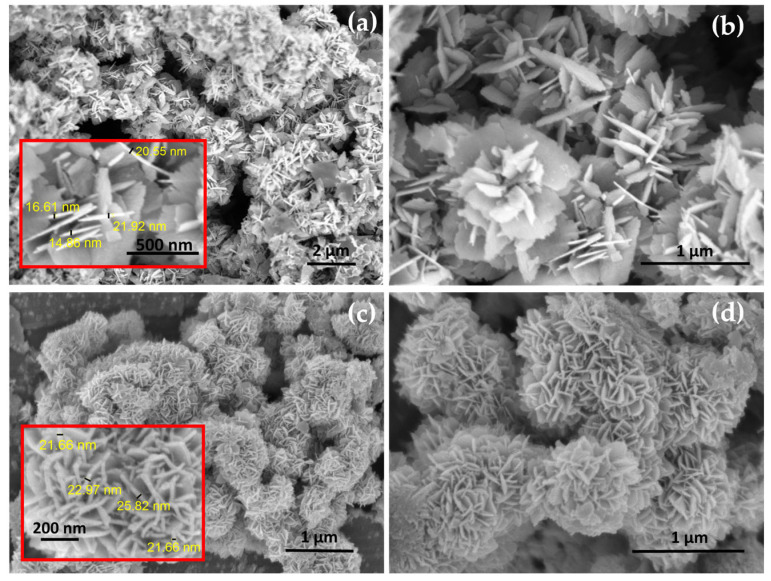
Microstructural features of the synthesized nanoparticles. Typical low and high-magnification FESEM images of as-synthesized (**a**,**b**) flower-like plates (INPs1) and (**c**,**d**) cauliflower-like nanoparticles (INPs2). The inserts indicate typical diameters of the platelets.

The morphologies of the as-synthesized INPs are characterized by a FESEM, as shown in Figure 1. Low and high-magnification FESEM images of INPs1 are presented in Figure 1a,b. It can be seen that they have flower-like plates; thus, INPs1 are referred to as flower-like plates in the following parts. FESEM images of INPs2 are given in Figure 1c,d. They show a cauliflower-like morphology with smaller and thinner platelets, which is why INPs2 are referred to as cauliflower-like nanoparticles in the following sections.

**Figure 2:**
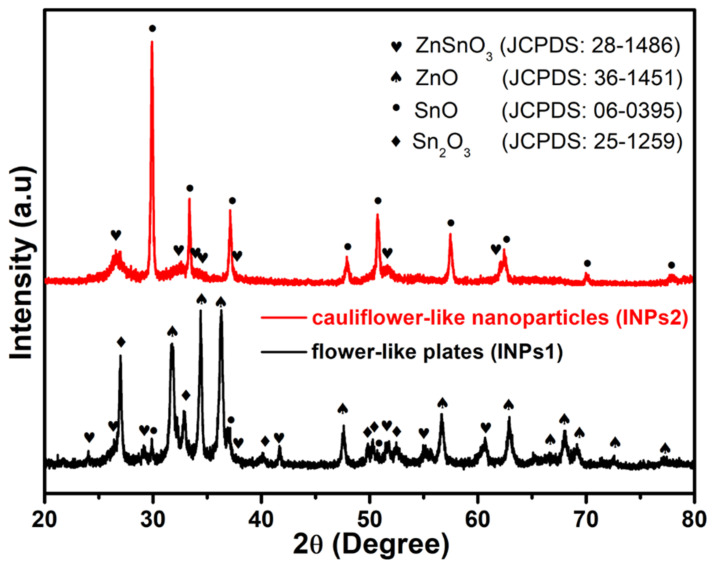
XRD diffraction patterns of as-synthesized nanoparticles (the black pattern designates flower-like plates (INPs1), and the red pattern designates cauliflower-like nanoparticles (INPs2).

XRD measurements were used to identify the crystal structure and the various phases of these synthesized nanocrystals. The phase patterns obtained are presented in Figure 2. The corresponding X-ray diffraction patterns of as-synthesized flower-like plates (INPs1) are indexed to orth-ZnSnO_3_ (JCPDS: 28-1486) and SnO (JCPDS: 06-0395), indicating that the SnO phase dominates the nanoparticles. The corresponding diffraction patterns of as-synthesized cauliflower-like nanoparticles (INPs2) contain mixed phases of orth-ZnSnO_3_ (JCPDS: 28-1486), sub-valent tin oxide species (Sn_2_O_3_, JCPDS: 25-1259); SnO, JCPDS: 06-0395); and ZnO (JCPDS: 36-1451), indicating that ZnO and Sn_2_O_3_ dominate in the structure of the nanoparticles.

**Figure 3:**
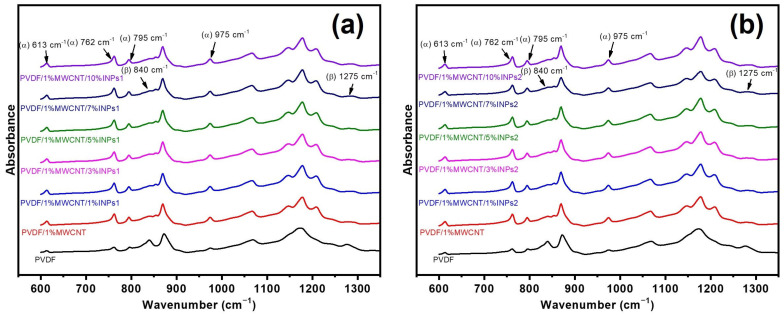
FTIR spectroscopy spectra results of pure PVDF, PVDF/1 wt% MWCNT, and PVDF/1 wt% MWCNT composites filled with different amounts of (**a**) flower-like plate nanocrystals (INPs1) and (**b**) cauliflower-like nanocrystals (INPs2).

Fourier-transform infrared spectroscopy (FTIR) was utilized to analyze the crystalline phases of the PVDF nanocomposites in the presence of nanocrystals and MWCNTs. The absorption bands at 613 cm^−1^, 762 cm^−1^, 795 cm^−1^, and 975 cm^−1^ are attributed to the characteristic bands of α-phase crystals, while bands at 840 cm^−1^ and 1275 cm^−1^ are assigned to the β-phase crystals [43]. Apparent bands at 613 cm^−1^, 762 cm^−1^, 795 cm^−1^, and 975 cm^−1^ assigned to the α-phase of PVDF emerge in the reference sample of PVDF and all the nanocomposites with MWCNTs and included nanocrystals, as seen in Figure 3. In addition, the absorption bands of the β-phase at 840 cm^−1^ and 1275 cm^−1^ can be observed in all nanocomposites based on PVDF/1 wt% MWCNT, independent of the amount and morphology of the nanocrystals. It has been confirmed that a strong interaction exists between the dipoles in −CF2 bands of PVDF and the π-electrons on the MWCNT surface, leading to fractional PVDF crystals transferring from the α-phase to the β-phase. Accordingly, the presence of the β-phase crystalline regions can be associated with interactions between PVDF and CNTs [25]. For both INP types, their introduction does not influence the FTIR spectra of PVDF/1 wt% MWCNT.

**Figure 4:**
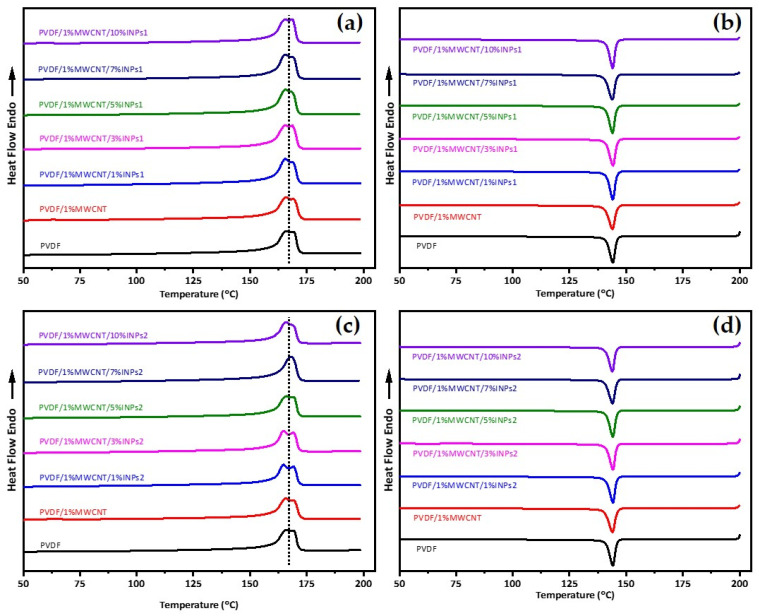
DSC heat flow curves of (**a**) first heating and (**b**) cooling for PVDF, PVDF/1 wt% MWCNT, and corresponding PVDF/1 wt% MWCNT with different amounts of flower-like plate nanocrystals (INPs1); (**c**) first heating and (**d**) cooling for corresponding PVDF/1 wt% MWCNT with different amounts of cauliflower-like nanocrystals (INPs2).

**Figure 5:**
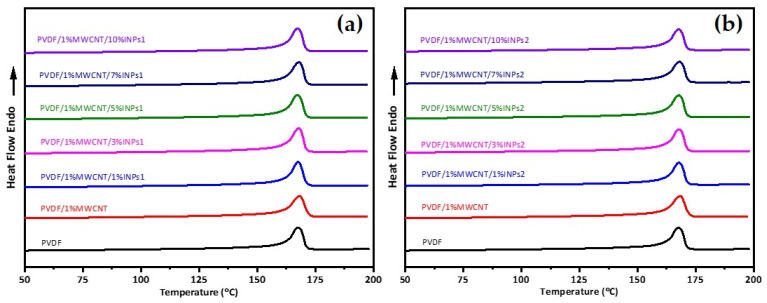
DSC heat flow curves of second heating for PVDF, PVDF/1 wt% MWCNT, and corresponding PVDF/1 wt% MWCNT composites with different amounts of (**a**) flower-like plate nanocrystals (INPs1) and (**b**) cauliflower-like nanocrystals (INPs2).

DSC was used to analyze the effect of MWCNT and synthesized nanoparticle addition on PVDF crystallization and evaluate their interactions. The corresponding results for the first heating and subsequent cooling curves from DSC are represented in Figure 4. It is observed that adding INPs to the PVDF/MWCNT composite does not lead to changes in the melting point and crystallization peak temperatures of PVDF. This phenomenon can be observed for both added INP types with different morphological features and phase characteristics. Furthermore, interestingly, this is also observed for all studied amounts of INPs. Therefore, adding synthesized INPs does not affect the crystallization and melting behavior of the PVDF composite. The second heat flow curves are given in Figure 5 and appear identical to the DSC results of the first heating.

**Figure 6:**
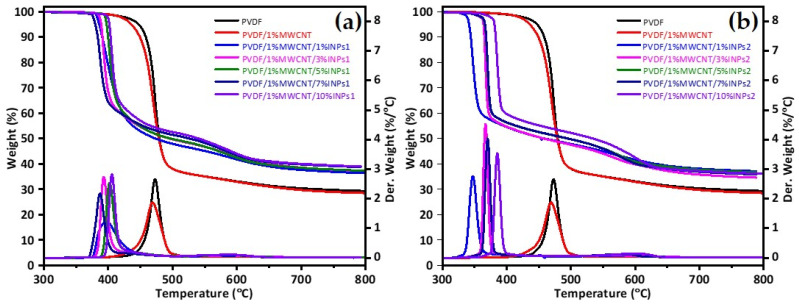
Thermogravimetric analysis (TGA) of PVDF, PVDF/1 wt% MWCNT, and corresponding PVDF/1 wt% MWCNT composites with different amounts of (**a**) flower-like plate nanocrystals (INPs1) and (**b**) cauliflower-like nanocrystals (INPs2).

TGA was used to evaluate decomposition temperatures and, eventually, the stability of the produced samples in a specific temperature range. The TGA results in Figure 6 show that for both nanocomposites with different types of nanocrystals, the decomposition temperature was lower than that of pure PVDF and the PVDF/1 wt% MWCNT composite. It is observed that the addition of cauliflower-like nanoparticles (INPs2) significantly shifts the degradation of PVDF to lower temperatures, as seen in Figure 6b. The nanocomposites with 1 wt% INPs2 show the most significant influence, with a 120 K lower maximum degradation temperature than pure PVDF. In addition, this nanocomposite degrades slower than the samples with higher INPs2 amounts. However, it is seen that the maximum temperature of degradation increases again as nanoparticle content increases. The nanocomposite with 10 wt% nanocrystal INPs2 shows only a 100 K lower maximum degradation temperature than PVDF.

As seen in Figure 6a, adding flower-like nanocrystals (INPs1) to PVDF shifts the degradation temperature of the nanocomposites to lower temperatures, but not as much as the addition of cauliflower-like nanocrystals (INPs2). The nanocomposite with 1 wt% nanocrystal INPs1 shows significantly different degradation behavior than those with higher contents. Degradation occurs very slowly, with a maximum of 394 °C, compared to other samples. The nanocomposites with higher nanocrystal INPs1 contents degrade again at the same rate as PVDF. However, the maximum decomposition temperature changes with INPs1 content are not linear, as nanocomposites were observed with the cauliflower-like nanoparticle (INPs2). These results indicate that adding synthesized INPs to a PVDF matrix significantly changes its thermal properties. It is very complex to understand how inorganic nanoparticles affect the thermal stability of PVDF composites because this effect is related to various factors, such as the type of INPs, the structure of the composites, and the interaction between the PVDF and MWCNT components [44]. The morphology, surface area, phases included, and crystallite size of the nanoparticles may change the interaction of the synthesized INPs with PVDF. Consequently, it is assumed that the changes in the thermal behavior of the produced nanocomposites result from combining all these influencing factors.

**Figure 7:**
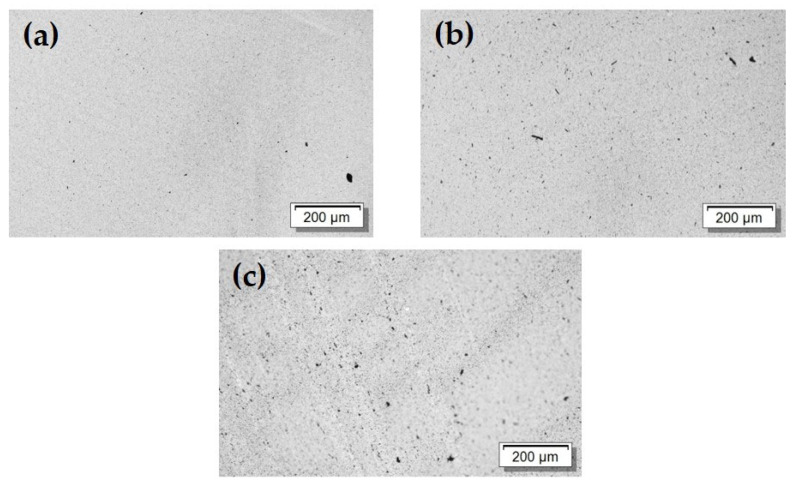
Transmission light microscopy images (section thickness 5 μm) (**a**) PVDF/1 wt% MWCNT; (**b**) PVDF/1 wt% MWCNT/3 wt% INPs1; (**c**) PVDF/1 wt% MWCNT/3 wt% INPs2.

Light microscopy was used to study the MWCNT dispersion and distribution in the composites. The images (Figure 7) show the remaining non-dispersed MWCNT agglomerates, including some larger black dots and many very small dark spots. The added INPs1 and INPs2 are not visible in transmitted light due to the optical transparency. However, it is observed that there is a slightly higher amount of visible dark areas in the composite structures with the INPs, especially when using INPs2. This indicates a slightly poorer CNT dispersion after adding INPs.

**Figure 8:**
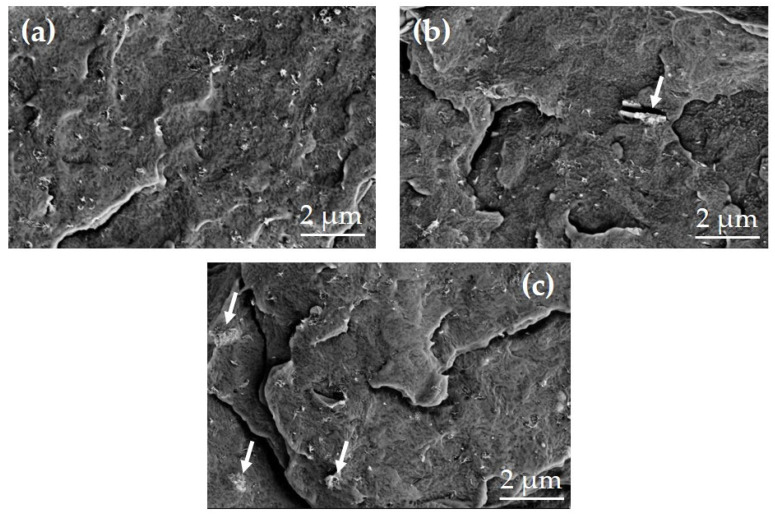
SEM images of the cryo-fractured surfaces of (**a**) PVDF/1 wt% MWCNT composite and PVDF/1 wt% MWCNT with 3 wt% of (**b**) INPs1 or (**c**) INPs2 (arrows indicate parts of the INPs).

The microstructural features of the nanocomposites produced were analyzed using SEM. The structures of all the nanocomposites produced appear to be homogeneous, as can be seen in Figure 8. Some nanotubes protrude from the fractured surface, which looks similar in all three images. No large agglomerates originating from the added INPs can be recognized. It can therefore be assumed that the micrometer-sized structures seen in Figure 1 are at least partially broken into the larger platelet structures consisting of INPs1. One of these platelets of INPs1 can be seen in Figure 8b, while Figure 8c shows some small cauliflower-like segments of INPs2 marked by arrows.

**Figure 9:**
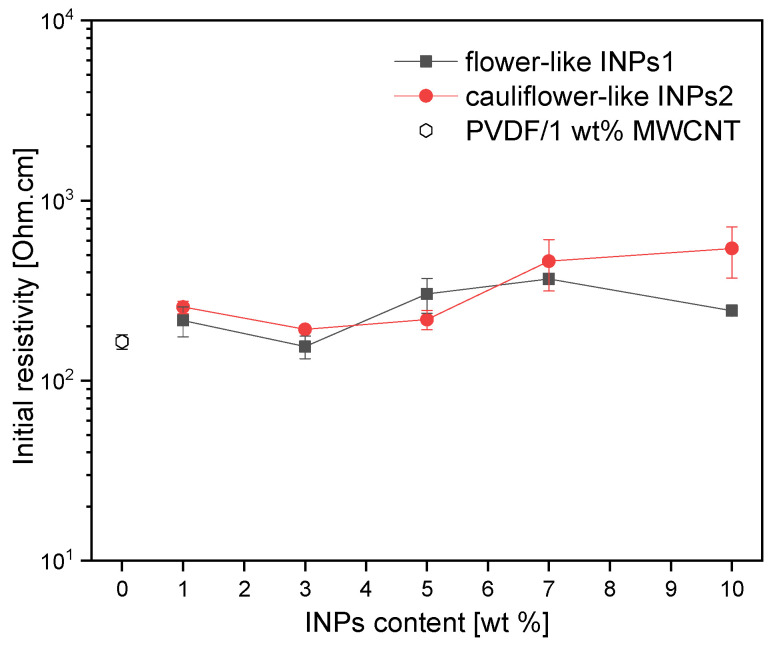
Changes in the initial electrical resistivity of PVDF/1 wt% MWCNT composites after embedding different amounts of inorganic nanoparticles.

Figure 9 illustrates the influence of incorporating nanocrystals, ranging from 1 to 10 wt% in concentration, on the initial electrical resistivity of PVDF/1 wt% MWCNT composites. Incorporating nanocrystals can be interpreted as a mechanism to slightly obstruct the percolation pathways of CNTs, thereby enhancing interfacial polarization. This phenomenon is more pronounced by INP crystals exhibiting cauliflower-like nanostructures (INPs2).

**Figure 10:**
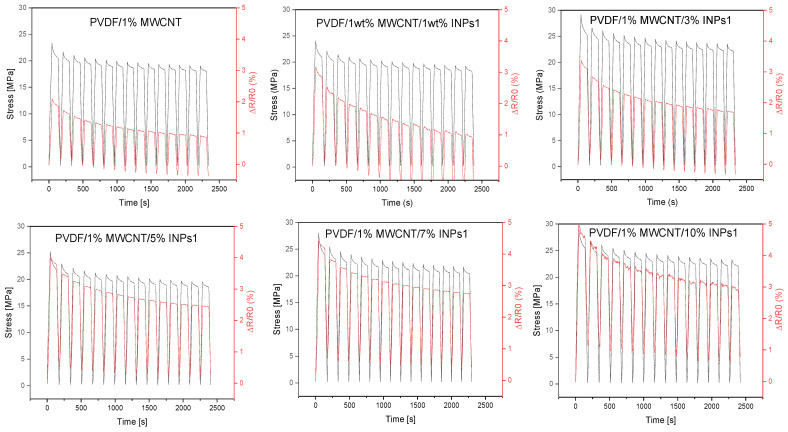
Results of cycling tests performed with 15 cycles up to 3% strain for PVDF/1 wt% MWCNT composites with flower-like plates (INPs1) at different amounts (1–10 wt%).

**Figure 11:**
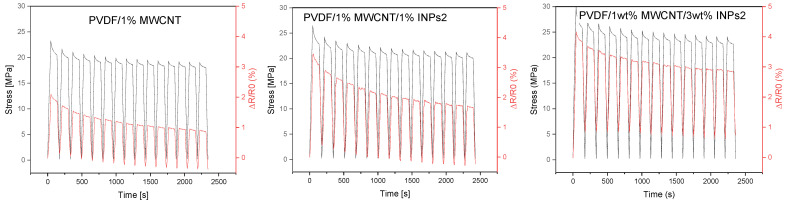

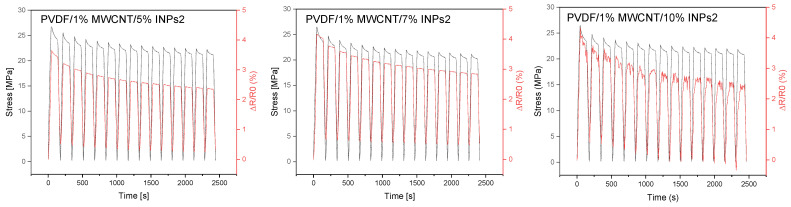
Results of cycling tests performed with 15 cycles up to 3% strain for PVDF/1 wt% MWCNT composites with cauliflower-like nanocrystals (INPs2) at different amounts (1–10 wt%).

In order to evaluate their piezoresistive behavior, the nanocomposites were subjected to cyclic strain sensing tests. The evaluation focused on strain sensitivity (measured as ΔR/R_0_) and cycle stability. The effect of the as-synthesized nanocrystal was evaluated relative to the reference composite (PVDF/1 wt% MWCNT). The 15 cycles tested for all composites were performed up to 3% strain, which is below the plastic deformation range of this composite, as known from previous research [45]. The results of the corresponding cyclic testing of composites, which can be assumed to have approximately similar initial resistances (see Figure 9), are presented in Figure 10 and Figure 11.

The dependence of the developed stress and the relative resistance change ΔR/R_0_ in the 15 strain-sensing cycles of the polymer composites obtained by adding flower-like plates (INPs1), containing predominantly ZnO and Sn_2_O_3_ phases, to the PVDF/1 wt% MWCNT composite at different amounts (1–10 wt%) are presented in Figure 10. The effect of stress relaxation is observable in the resistance change (ΔR/R_0_) curves of all composites between the stretched and relaxed states when holding the specimen for 90 s. All PVDF/1 wt% MWCNT composites containing INPs1 show significantly higher strain sensitivity than the PVDF/1 wt% MWCNT composite at an applied maximum strain of 3%. For all the examined samples, the maximum and minimum values of ΔR/R_0_ corresponding to the stretched and non-stretched states gradually decreased with increasing cycle numbers. The effect of decreasing or increasing values of the relative resistance change with the number of cycles has already been described in previous studies. It can be explained by the incomplete reconstruction of the conductive network during cyclic loading and unloading or by partial irreversible network changes and breakage [25,46], as well as the viscoelastic nature of the PVDF matrix. However, it is seen that the declining trend of ΔR/R_0_ values weakens with increasing INPs1 content in the PVDF/1 wt% MWCNT composites. In addition to that, the maximum and minimum values of ΔR/R_0_ increase with an increasing percentage of INPs1.

Cyclic tests on the polymer composites with cauliflower-like INPs2, containing predominantly SnO and orth-ZnSnO_3_ phases, in the PVDF/1 wt% MWCNT composites with different amounts (1–10 wt%) are presented in Figure 11. As with the INPs1-containing composites, all composites with INPs2 show a higher strain sensitivity than the PVDF/1 wt% MWCNT composite at the same maximum strain of 3%. While the peak and lowest values of ΔR/R_0_ for composites with INPs1 decrease significantly with increasing cycles, the decrease in ΔR/R_0_ values in both the stressed and relaxed states is not as pronounced for composites with INPs2.

**Figure 12:**
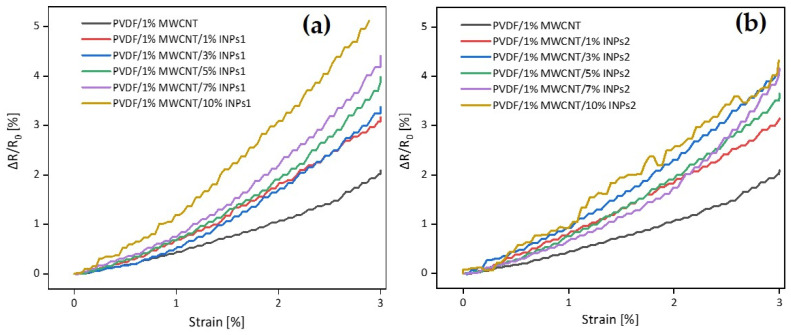
Comparative analysis of strain-induced electrical resistivity changes in the first straining period in PVDF/1 wt% MWCNT composites with different amounts of (**a**) flower-like plate nanocrystals (INPs1) and (**b**) cauliflower-like nanocrystals (INPs2).

Figure 12 illustrates the changes in electrical resistivity ΔR/R_0_ vs. strain for the straining of the first cycle of each nanocomposite. It is evident from the figure that adding flower-like plate nanoparticles (INPs1) into the composite improves the piezoresistive behavior slightly more than adding cauliflower-like nanoparticles (INPs2). These results demonstrate that adding these kinds of INPs can be used as an efficient method to improve the piezoresistive behavior of PVDF/MWCNT-based nanocomposites.

**Figure 13:**
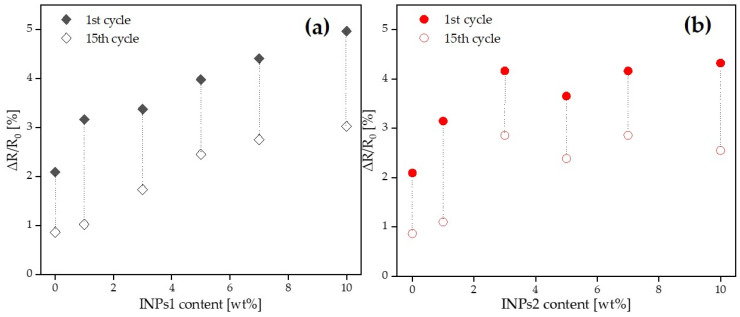
Variation of ΔR/R_0_ values in PVDF/1 wt% MWCNT composites embedded with different ratios of (**a**) flower-like plate nanocrystals (INPs1) and (**b**) cauliflower-like nanocrystals (INPs2) at 3% strain for the initial and final strain cycles.

Figure 13 summarizes the variation in the maximal ΔR/R_0_ values achieved after the loading to 3% for PVDF/1 wt% MWCNT composites with INPs1 and INPs2 after the initial and final loading. The summarized plot again shows that ΔR/R_0_ increases more strongly with the INP content after the first loading for INPs1 and that the absolute decrease in ΔR/R_0_ after the 15th loading is on average more pronounced for composites with INPs1 than for those with INPs2.

**Figure 14:**
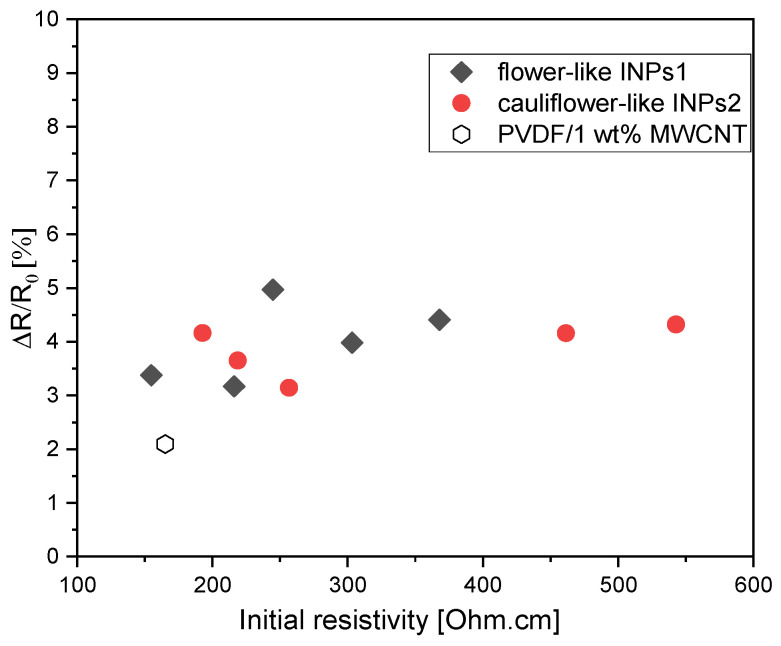
Dependence of piezoresistive behavior (relative resistance change after the straining in the 1st cycle) on the initial electrical resistance of PVDF/1 wt% MWCNT composites with INPs1 and INPs2.

The question of whether the higher strain sensitivity of the composites with INPs1 is related to the slight differences in the initial resistivity between both composite sets (see Figure 9) may arise. It is well known that composites with filler contents closer to the electrical percolation threshold, i.e., with a higher initial resistivity, have a higher sensing response [47]. To prove that, in Figure 14, the measured relative resistance change after the straining part in the 1st cycle was plotted vs. the initial resistivity of all samples. Noticeably, the ΔR/R_0_ values of all composites with INPs are above the value of the PVDF/1 wt% MWCNT composite, which has nearly the lowest initial resistance. However, between nanocomposites containing INPs1 and INPs2, there is no significant difference in the ΔR/R_0_ values vs. initial electrical resistance. The two composites with INPs2, which have the highest initial resistivities, do not show the highest sensing sensitivity. The composite with 10 wt% INPs1 exhibited the highest sensitivity despite having an intermediate initial resistivity among all samples. This indicates that the higher piezoresistive response in composites with INPs1 is not due to higher initial resistivity values but to a slightly altered network structure of the conductive network, which is influenced by the addition of the platelet-like nanostructures and is more susceptible to strain changes.

## 4. Conclusions

A strategy of adding inorganic semiconductor nanocrystals has been reported to fabricate PVDF/MWCNT-based nanocomposites to improve the strain sensitivity of strain sensors. The composites having 1–10 wt% of the inorganic nanocrystals and 1 wt% of MWCNTs could be prepared by melt mixing without processing problems and showed morphologies with good filler distribution and relatively homogenous structure.

Both series of composites, either with flower-like plates (INPs1) or with cauliflower-like nanoparticles (INPs2), show in cyclic strain sensing with 15 cycles up to 3% strain a higher piezoresistive sensitivity than the base PVDF/1 wt% MWCNT nanocomposite. A declining trend of ΔR/R_0_ values with increasing cycle numbers can be observed in all nanocomposites. In addition, stress and resistance relaxation were observed during the holding period before the strain’s release. This indicates that the network structure changes are not fully reversible. Furthermore, the maximum and minimum values of ΔR/R_0_ after each cycle part tendentially increase with the amount of nanocrystals. Adding INPs1 to the composite improved the piezoresistive behavior slightly more than adding INPs2. After the straining in the first cycle, the change in relative resistance was 2.5-fold for nanocomposites with INPs1 and 2.0-fold with INPs2 relative to the PVDF/1 wt% MWCNT composite.

The analysis of initial electrical resistivities and piezoresistivity measurements of all fabricated composites showed that the integration of nanocrystals resulted in a slight increase in electrical resistivity compared to the PVDF/1 wt% MWCNT composite, which is more pronounced in the presence of INPs2 crystals. These nanocrystals, recognized as semiconductors, are assumed to interfere with the percolation pathways of CNTs, thereby enhancing interfacial polarization.

TGA results indicate that adding synthesized INPs to the PVDF matrix significantly reduces its thermal stability. The decomposition temperatures are lower than those of pure PVDF and PVDF/1 wt% MWCNT for both nanoparticle-added composites with different nanocrystals. In addition, it is observed that adding INPs2 to the PVDF/MWCNT nanocomposite leads to a higher reduction in the maximum degradation temperature of the nanocomposites relative to adding INPs1. It is thought that the changes in thermal behavior may result from the morphology, surface area, phases included, and crystallite size of nanoparticles. Therefore, the surface area is expected to be higher for the finer-structured INPs2 with the cauliflower-like morphology. However, the dependencies mentioned here require a more detailed examination.

Complex and adaptable composites enhanced with inorganic semiconductor nanocrystals have the potential for the fabrication of sensors that can be tailored to different applications. This opens up opportunities for future research to explore the correlation between the piezoresistive properties and the microstructure of conductive polymer composites. These possibilities open up exciting prospects for future studies in this field.

## Data Availability

Data are contained within the article.
